# A 3D-Printed Fin Ray Effect Inspired Soft Robotic Gripper with Force Feedback

**DOI:** 10.3390/mi12101141

**Published:** 2021-09-23

**Authors:** Yang Yang, Kaixiang Jin, Honghui Zhu, Gongfei Song, Haojian Lu, Long Kang

**Affiliations:** 1 School of Automation, Nanjing University of Information Science and Technology, Nanjing 210044, China; 201813930174@nuist.edu.cn (K.J.); 20201249181@nuist.edu.cn (H.Z.); gfsong@nuist.edu.cn (G.S.); 2 Key Laboratory of Advanced Control and Optimization for Chemical Processes, East China University of Science and Technology, Ministry of Education, Shanghai 200237, China; 3 Institute of Cyber-Systems and Control, State Key Laboratory of Industrial Control Technology, Zhejiang University, Hangzhou 310027, China; luhaojian@zju.edu.cn; 4 PCA Lab, Key Laboratory of Intelligent Perception and Systems for High-Dimensional Information of Ministry of Education and Jiangsu Key Lab of Image and Video Understanding for Social Security, School of Computer Science and Engineering, Nanjing University of Science and Technology, Nanjing 210094, China

**Keywords:** soft robotic gripper, force feedback, 3D printing, compliant structure, Fin Ray effect

## Abstract

Soft robotic grippers are able to carry out many tasks that traditional rigid-bodied grippers cannot perform but often have many limitations in terms of control and feedback. In this study, a Fin Ray effect inspired soft robotic gripper is proposed with its whole body directly 3D printed using soft material without the need of assembly. As a result, the soft gripper has a light weight, simple structure, is enabled with high compliance and conformability, and is able to grasp objects with arbitrary geometry. A force sensor is embedded in the inner side of the gripper, which allows the contact force required to grip the object to be measured in order to guarantee successful grasping and to provide the most suitable gripping force. In addition, it enables control and data monitoring of the gripper’s operating state at all times. Characterization and grasping demonstration of the gripper are given in the Experiment section. Results show that the gripper can be used in a wide range of scenarios and applications, such as the service robot and food industry.

## 1. Introduction

With the continuous development of robotics, traditional rigid and bionic robotic hands have been widely used in various fields [1], playing an important role in the mechanization and automation of industrial production, freeing humans from repetitive labor [2]. However, conventional robotic hands usually consist of rigid parts and rigid joints, which have low compliance and lack the ability to adapt to unstructured environments [3,4]. In addition, many conventional gripper ends are made of rigid materials and are in rigid contact with the object to be gripped, which requires that the object to be gripped is not fragile and deformable, which greatly limits the versatility and flexibility of a robotic gripper [5].

As society evolves, the requirements for robotic devices vary greatly from field to field, including safety during human–machine interaction, degrees of freedom, flexibility, and adaptability in unstructured environments [6], etc. For complicated grasping situations, a high degree of freedom is often required for mechanical grippers, which increases the cost of the structure and the control of such grippers [7]. As such, soft grippers made of flexible materials are developed for manipulating fragile or irregularly shaped objects [8].

In the gripping of objects where changes in shape are readily encountered, such as foodstuffs, irregular geometric objects, and biological tissues, high flexibility and adaptability of the manipulation device are required. Conventional rigid-bodied robotic grippers would need a delicate mechanism design and a complicated control strategy to perform these tasks [9]. The soft robotic grippers, on the other hand, will conform to the grasped objects’ shape and do not cause damage to the surface benefited from the gripper body’s inherent compliance [10,11].

The existing soft robotic grippers have two-fingered and three-fingered configurations. In contrast to the two-fingered configuration, the three-fingered configuration will put the object in a more balanced position and will have a larger contact surface area when holding an object. Therefore, we chose to use three fingers in our gripper design, which are equally distributed with the structure of the robotic fingers inspired by fish fins [12,13,14,15]. Taking use of the Fin Ray effect, the gripper can automatically wrap around the grasped object regardless of its shape. In terms of the actuation principle, there are two main types. In pneumatic or hydraulic, the soft robot gripper is built with hollow channels, and the flow of the liquid or gas inside the channels makes the gripper move [16,17]. The second actuation type is “smart material” such as shape memory alloy (SMA) [18,19], dielectric elastomer actuators (DEAs) [20], ionic polymer¬–metal composites (IPMC) actuators [21], etc., which deform in response to external stimuli to drive the robotic gripper. Actuation with a pneumatic or hydraulic system usually brings larger volume and requires auxiliary devices, such as an air compressor or a pump and valves, which make the whole robotic system bulky and noisy.

**Figure 1:**
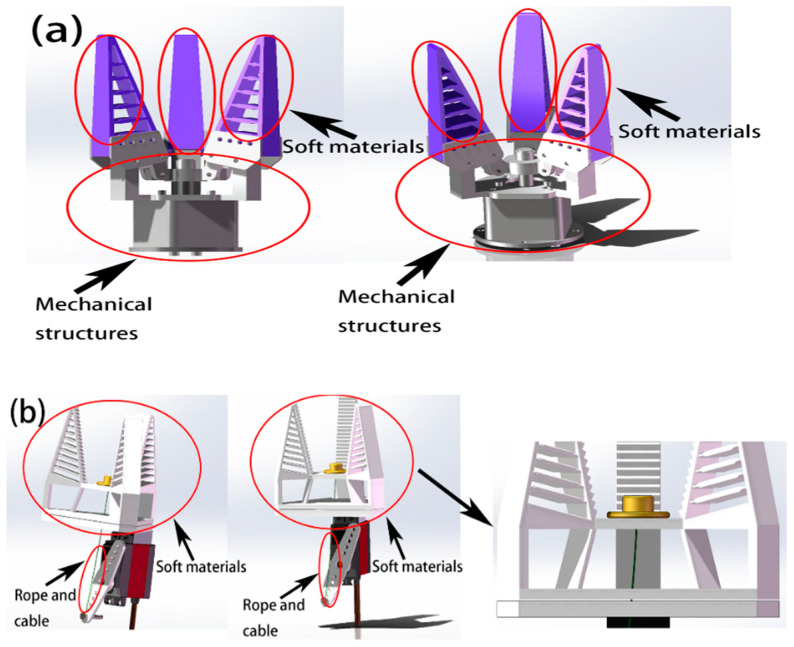
Comparison of a common commercially available Fin Ray effect inspired soft gripper with the soft gripper proposed in this paper. (**a**) shows a common commercially available soft adaptive gripper. (**b**) shows the soft gripper designed in this paper.

In this study, we used 3D-printing technology to fabricate the whole soft robotic gripper body in one piece, without the need for assembly of each finger, which greatly reduces the number of components and complexity of the system. The soft robotic gripper has a hollow structure in the middle with an upper plate connecting the three fingers. By applying force to the center of the upper plate, the three fingers will bend rather than having to drive each finger individually. This saves on the cost of the actuator and reduces the overall size even further, making the gripper more compact. Figure 1 shows the comparison between a commercially available Fin Ray effect inspired gripper (Three-fingered Compliant Bionic Gripper, Wheeltec Intelligent Technology, Dongguan, China [22]) in Figure 1a and the soft gripper designed in this paper in Figure 1b.

In comparison, the soft gripper commonly found on the market that is shown in Figure 1a has a complex mechanical structure, where each finger of the gripper needs to be fixed and driven by a separate mechanical structure, making it geometrically heavier than the gripper designed in this paper, and more complicated to fabricate and install, increasing production and time costs. In contrast, the soft gripper proposed in this paper that is shown in Figure 1b has one monolithic gripper body, 3D printed from soft material, which requires no extra parts or fixing bases and is simple to equip. Table 1 illustrates the differences between the proposed design of a flexible gripper and the commercially available gripper in [22].

For some fragile objects to be grasped, we want to be able to control the amount of force applied by the robotic gripper and be able to observe and make adjustments in time [23]. Juan et al. mounted a tactile sensor on Fin Ray effect inspired flexible adaptive grippers and aimed for an object recognition task based on deep convolutional neural networks [24]. Data acquisition electronics are indispensable, and a certain amount of computing power is required to process the acquired data. In this study, we have integrated a pressure sensor embedded in the inner side of the robotic finger to give feedback on the force applied by the soft gripper, which we can observe in real time on a serial monitor. The control side of the robot is connected to a Bluetooth module, allowing us to control the soft robot in real time from a device with a Bluetooth transceiver. Highlights of this work are as follows:

(1) A monolithic 3D-printed soft adaptive gripper based on Fin Ray effect is proposed with ease of fabrication;
(2) A pressure sensor is integrated in the soft gripper to realize force feedback during grasping;
(3) Experiments are conducted to characterize the gripper and to demonstrate the improved grasping performance with force feedback.

The rest of this paper is organized in the following manner: Section 2 presents the design principle of the proposed gripper. In Section 3, force feedback control strategy and characterization of the gripper are given firstly, followed by the grasping experiments. Lastly, Section 4 concludes the paper and discusses potential future work.

## 2. Design and Analysis

**Figure 2:**
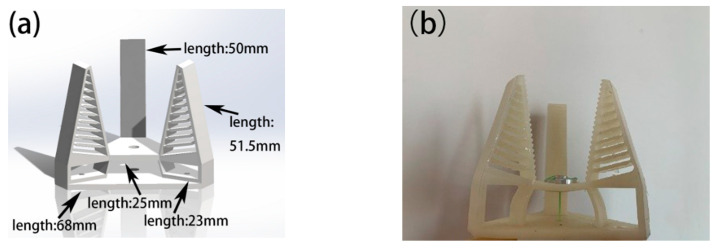
Conceptual drawing of a 3D-printed Fin Ray effect inspired soft robotic gripper: (**a**) 3D model; (**b**) real prototype.

The whole part of the soft robotic gripper body is 3D printed in one piece, which makes it easy and cost effective to produce the gripper. In this design, the body of the soft gripper was 3D printed by a ZRapid Tech model iSLA 660 light-curing 3D printer using Formlabs elastic resin material. The material has a modulus of elasticity of 0.8 kgf/mm^2^ and a hardness of 40 Shore A. The model (a) and the actual gripper (b) are shown in Figure 2.

The fish fin structure allows the robotic gripper to be more adaptive and relatively stable when gripping objects, allowing it to cope with different objects with ease [25]. The characteristics and advantages of the Fin Ray structure have been systematically investigated in previous studies with structural modeling, calculations, and experiments [14,26]. Therefore, we have adopted the Fin Ray structure as well as the inner patterns. The fins are distributed diagonally parallel to the fingers and are connected by an elastic structure. The hollow structure between the fins, which can be flexibly altered by external forces, allows the finger to fit more closely to the object when grasping it, improving the wrapping of the object and providing protection. In this design, we set the initial tilt of the gripper fingers at 30 degrees and the finger length at 50 mm. There are tiny raised triangular structures evenly distributed on the inner side of the fingers, which can effectively increase the friction between the fingers and the object being gripped, preventing accidents such as falling off when gripping some smooth objects. The robotic gripper has three fingers distributed in a circular sequence. Compared to the two-finger structure, the three-finger robotic gripper is more stable and reliable and can be adapted to more scenarios.

For the actuation of the gripper, force applied to the center of the gripper base’s upper plate will lead to bending movements of the gripper. When force is not applied, the deformed fingers will resume their initial position due to the elasticity of the soft materials that they are composed of. The external force is applied by a traction wire, which connects the rotating fittings of the servo motor to the upper central part of the robotic gripper. The servo motors can be of different types depending on the grasping scenarios. In our design, one actuator can drive all three fingers, which saves both actuation cost and overall weight. Changes in the point of application of force can indirectly change the movement of the robotic gripper to cope with different objects.

**Figure 3:**
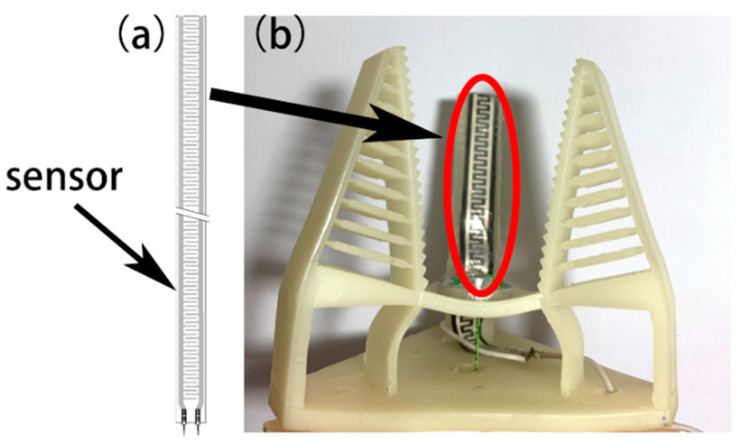
(**a**) Pressure film sensor. (**b**) Gripper with embedded sensor.

**Figure 4:**
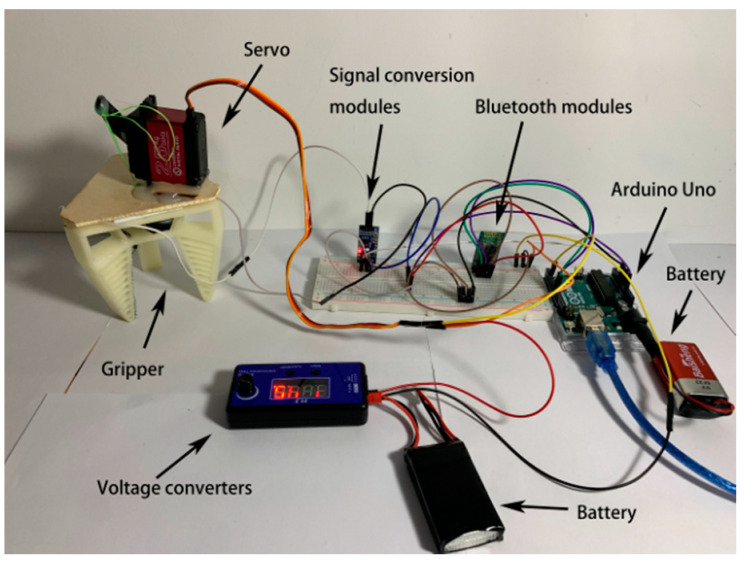
The integral soft robotic gripper system.

In order to satisfy the needs of monitoring the contact force or making adjustments to the force applied by the gripper under some conditions, a patch-type thin-film pressure sensor, which is essentially a piezoresistive sensor, was built into the inside of the robotic gripper’s finger (only one sensor was applied in this study). Like the gripper body, the thin-film pressure sensor is also compliant and deformable, as shown in Figure 3a. It was embedded in the finger using Smooth-On’s Ecoflex silicone, and the two can be satisfactorily combined, as shown in Figure 3b. The sensor was 0.25 mm thick and can be adequately fitted on the inside of the finger. The thin-film pressure sensor was connected to a voltage conversion module to form a complete system to drive the entire robotic gripper, as shown in Figure 4.

On the back of the flexible gripper, there is a fixed bracket on which the actuation system, such as the servo motor in Figure 4, is mounted. When the servo motor receives a signal, it starts to perform the corresponding action to drive the pulling wire. Here, we used a DS3225 servo motor from DSSERVO with a torque of 25 kg and a servo weight of 60 g. There is an overhead structure between the base of the gripper and the upper plate, which, due to the wire pulling, causes the space between them to change so that the fingers bend. The height of this overhead structure was 2.3 cm. The control section consisted of a microcontroller and a Bluetooth controller; here, we used the HC-05 Bluetooth module with a Bluetooth 4.0 interface. The microcontroller can be of different types and models depending on the needs; here, we used the Arduino Uno as a sample core processor and connected the Bluetooth module to a pressure sensor (Style No.: RP-L TDS REV C, Shenzhen K-CUT Inc.). When the Arduino Uno receives the feedback signal, it processes the signal and makes the corresponding action. The battery connected to the Arduino is 5 V and the battery connected to the servo is 8 V.

**Figure 5:**
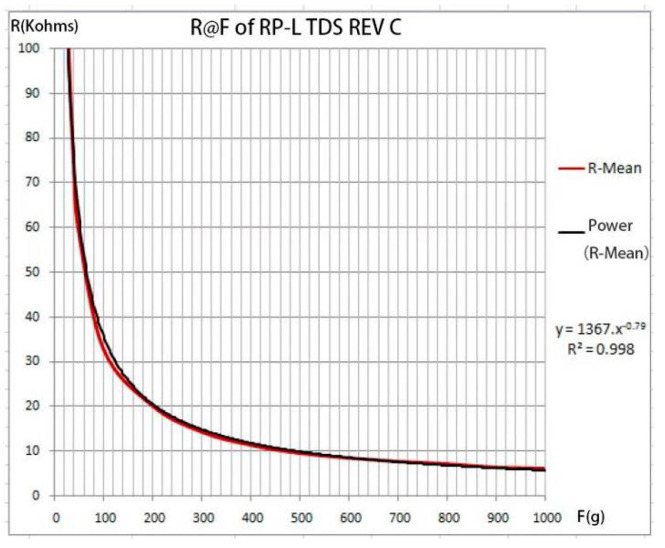
Pressure sensor calibration.

The flexible thin-film pressure sensor (Style No.: RP-L TDS REV C, Shenzhen K-CUT Inc.) applied in this study consisted of a polyester film with good compliance and highly conductive material as well as pressure sensitive material. When pressure is applied to the sensing area, the lower disconnected layer conducts through the sensitive layer and the output value of the port resistance varies according to the pressure. Because the state of the robotic gripper changes with time when it is in operation, the sensor needs a short response time if more accurate feedback is to be obtained. The activation time of the thin-film pressure sensor is less than 0.01 s to roughly meet the application of the robotic gripper in most scenarios, and its durability is long-lived. Before the sensor is put into use, it has to be calibrated. Figure 5 shows the relationship between the change in the sensor’s resistance with applied pressure in a particular test environment. The red lines are actual data while the black lines are fitted curves.

## 3. Experiment

**Figure 6:**
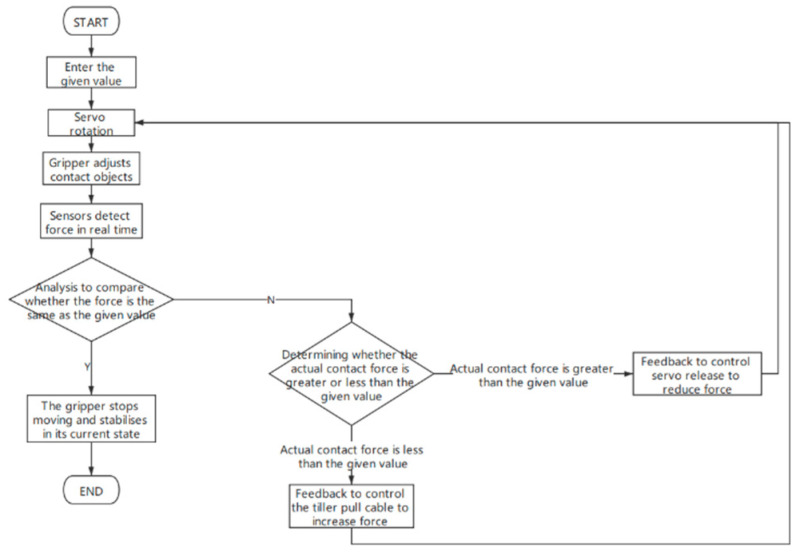
Workflow diagram of the proposed soft robotic gripper with force feedback function.

In the Experiment section, the force feedback system was relied upon to measure contact force for various grasping tasks. Figure 6 presents the workflow diagram of the proposed soft robotic gripper with force feedback. The system consists of the following components: an Arduino Uno controller, a servo motor for pulling wire and actuating the gripper, a power supply, a force sensor signal conversion module, and a Bluetooth module. During operation, the serial port of the Arduino Uno was used to receive input from the PC or the command sender and converted it into commands. When a given value is entered from the PC or the command sender, the servo rotates according to the predetermined expectation, the wire is pulled, shortening the distance between the upper and lower plates of the gripper, and the fingers move accordingly. The pressure sensor detects the contact force between the finger and the grasped object in real time. At each moment, the servo acts according to the comparison result between the measured force value and the predetermined value. If the measured force is smaller than the given value, then the controller will make the servo pull the cable to increase the contact force. Conversely, the servo will be controlled to release the cable in order to reduce the contact force. The servo will stop, and the gripper will stabilize when the measured force is the same with the given value, as shown in Figure 6.

### 3.1. Characterization of the Gripper

**Figure 7:**
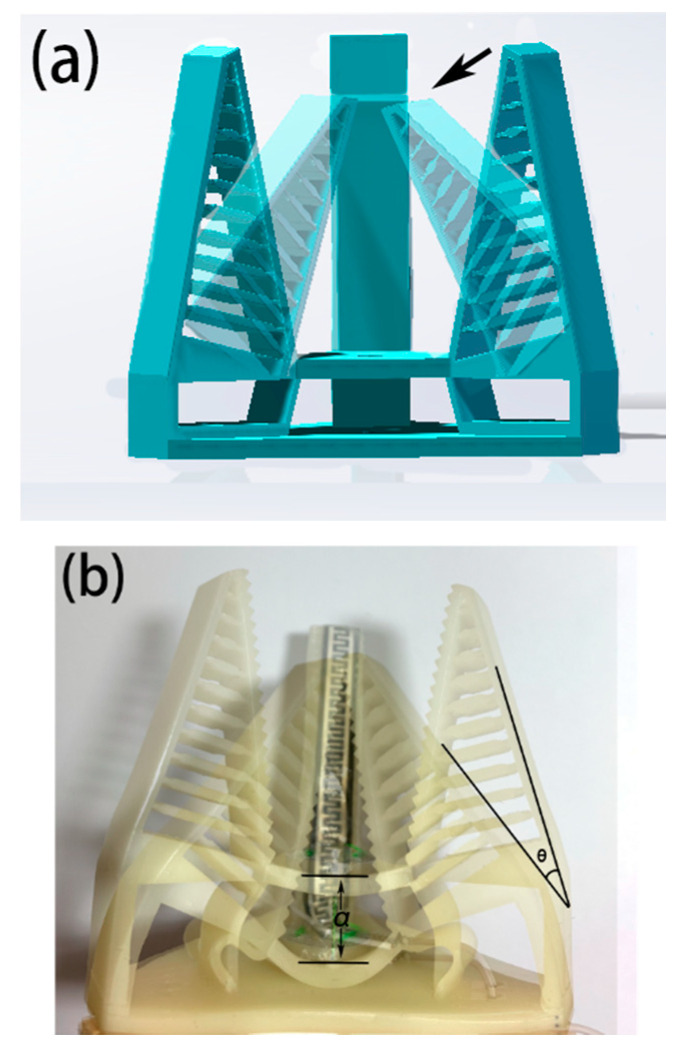
Schematic diagram of the angular displacement of the finger end θ and the length of the pulling wire movement. (**a**) shows the displacement schematic in the modeling and (**b**) shows the physical drawing, where *α* is the change in displacement length of the pulling wire and *θ* is the change in angular displacement of the end of the finger.

**Figure 8:**
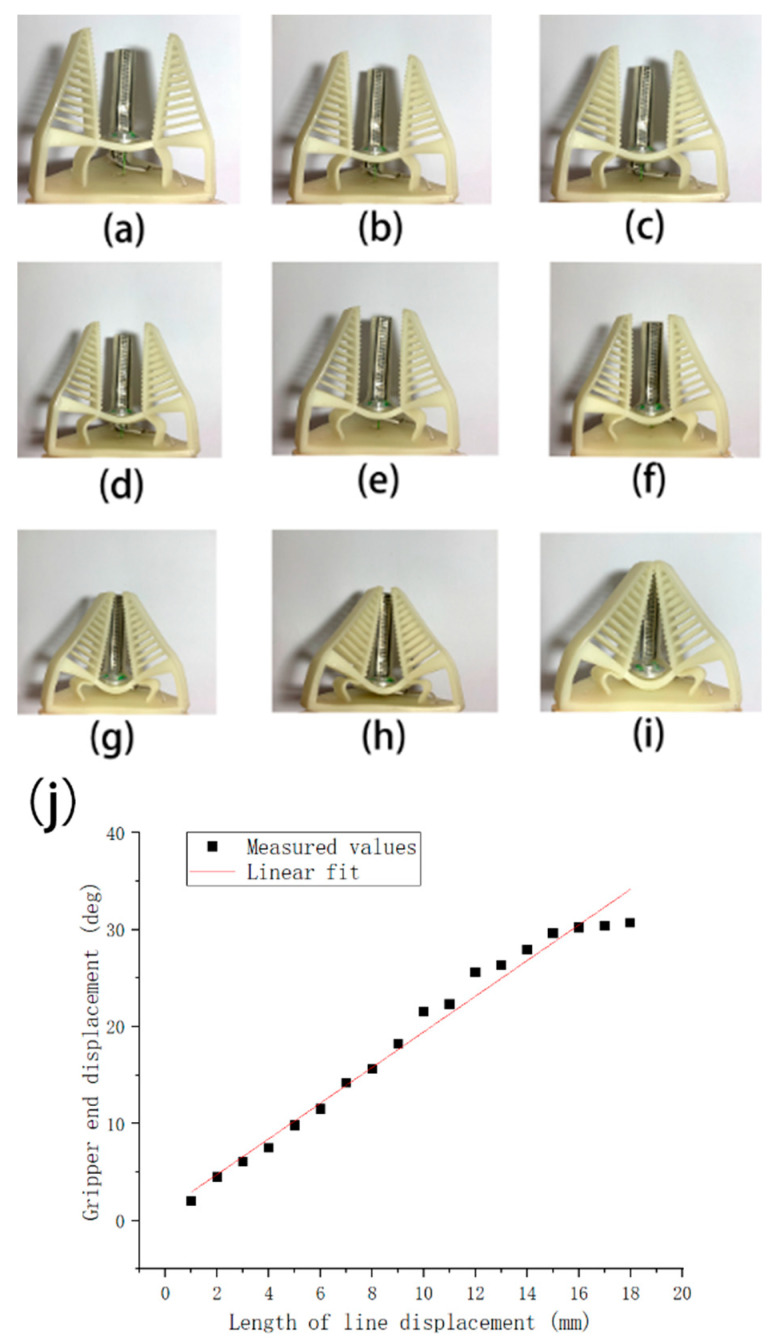
Changes in the state of the gripper during gripping with different degrees of deformation as shown in (**a**–**i**). The finger end angle change was measured once for every 1 mm change in the displacement of the pulling wire during the experiment, and a total of 18 numerical samples were taken. (**j**) shows the degree of deformation of the end of the finger in relation to the change in the displacement of the pulling wire.

To characterize the gripper, the relationship between the angular displacement of the finger end θ (as shown in Figure 7) and the length of the pulling wire movement α need to be investigated. In this experiment, marks were made at 1 mm intervals on the gripper’s pulling wire, and the original state of the gripper was used as the origin to observe and record the angular deflection of the end of the finger. The finger’s angular deflection was recorded by a HD camera and the test result is presented in Figure 8. From the test result, the angular deflection of the finger increased as the displacement of the pulling wire increased. We conducted linear fitting of the measured data, and the linear fit formula is:

*θ* = 1.84*α* + 1.10 (1)

**Figure 9:**
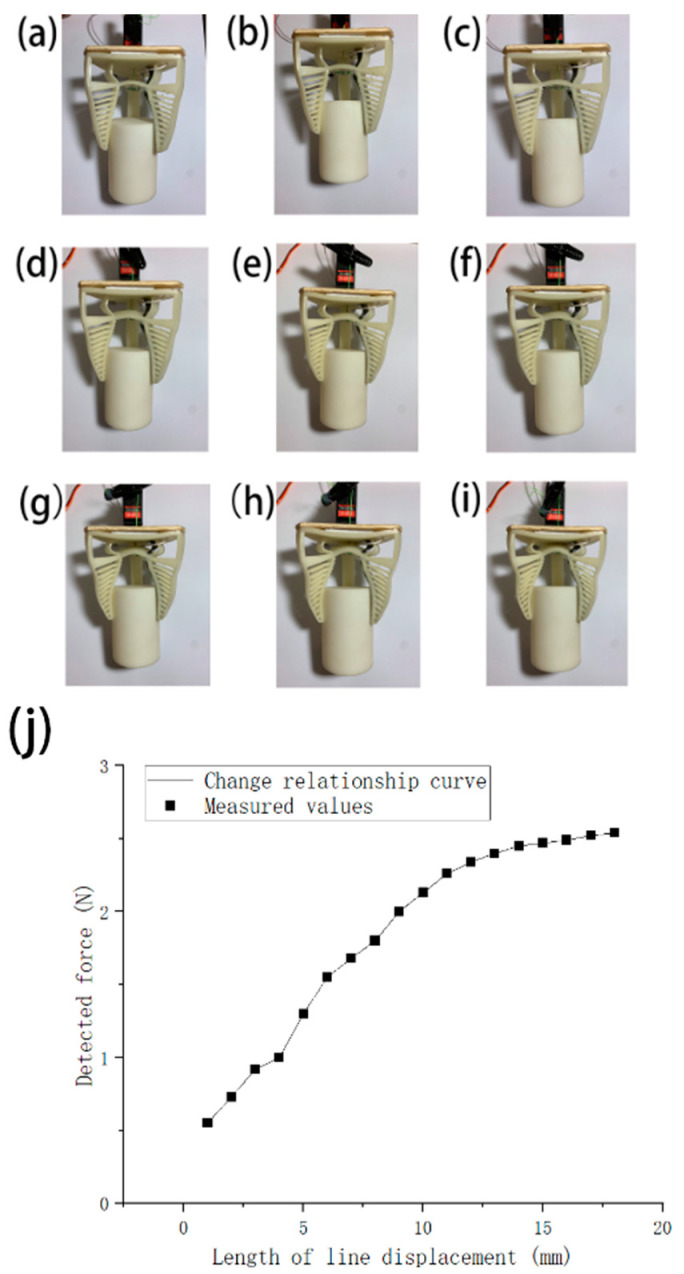
Force measurement when grasping an object. (**a**–**i**) are snapshots of grasping a 3D-printed cylinder. (**j**) shows a graph of the relationship between the change in force during grasping of an object and the change in displacement of the pulling wire.

Next, the soft gripper’s force feedback system was investigated. The values of the gripping force collected by the sensor were inputted to the Arduino Uno controller and can be read through the serial monitor or external display at the current moment. The relationship between the measured force values and the variation of the displacement of the pulling wire was investigated in this experiment. A hollow cylinder 3D-printed using ABS material was used as the gripped object with a weight of 200 g (diameter: 50 mm, height: 70 mm), as shown in Figure 9.

From the experimental result in Figure 9, the gripper fits the cylinder profile and reaches about 2.4 N gripping force when the pulling wire is displaced by 13 mm. Consequently, the gripping force and finger deformation both tended to be smooth and did not fluctuate excessively even though the servo motor continued to pull the wire.

### 3.2. Wrap-Around Grasping

**Figure 10:**
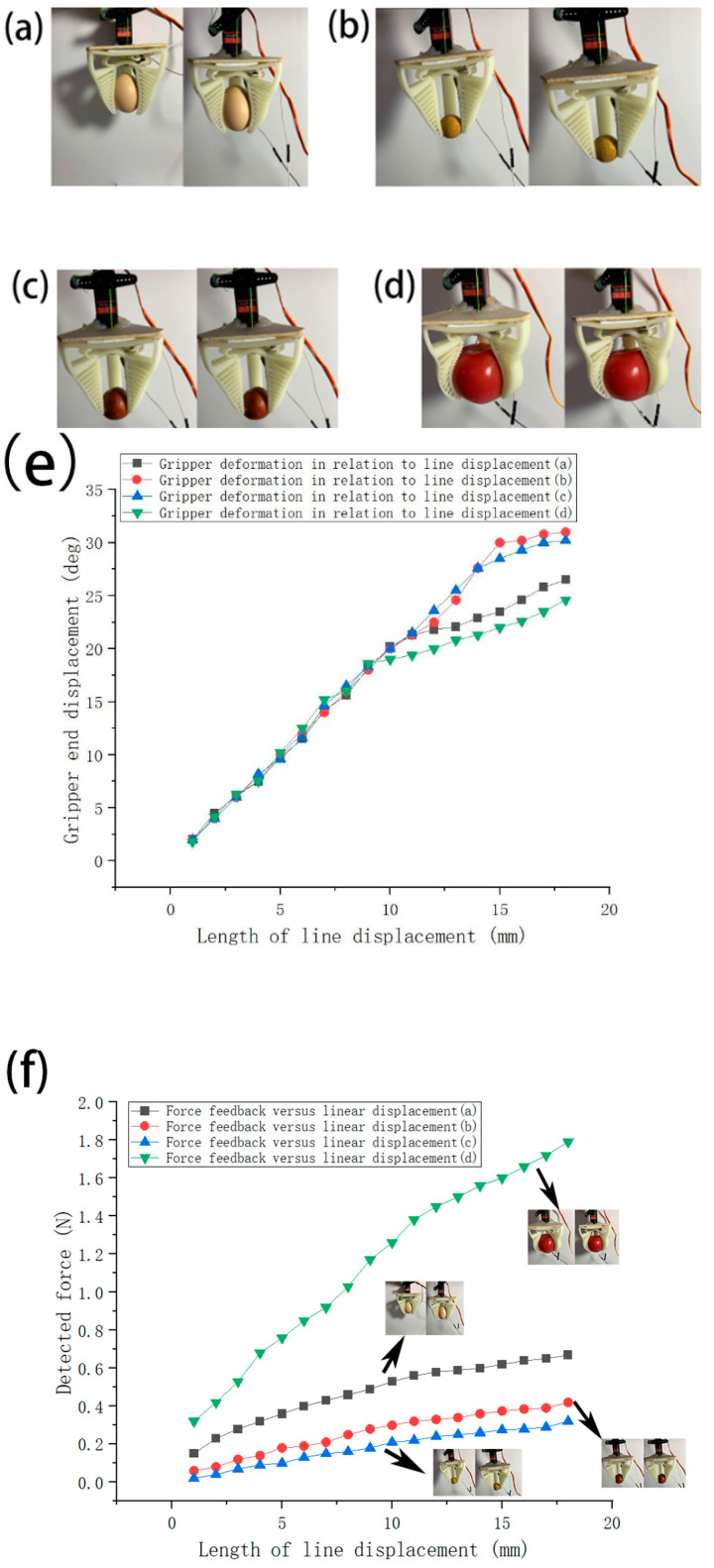
Examples of grasping objects (**a**–**d**). The graphs show examples of grasping postures of the force feedback gripper in grasping raw eggs (**a**), longans (**b**), dates (**c**), and tomatoes (**d**). (**e**) shows a graph of the variation in the degree of deformation of the pulling wire versus the end of the finger for the four sample experiments (**a**–**d**). (**f**) shows the variation of the pulling line versus force feedback for the four sample objects (**a**–**d**).

In this test, the gripper end angular displacement and force feedback’s variation with displacement of the pulling wire were tested for objects with different sizes and shapes. For demonstration, common life objects, such as raw eggs, longans, dates, and tomatoes were used as experimental samples for comparison and analysis, as shown in Figure 10.

Through the above experiments, we observed that the force exerted by the gripper on an object increased linearly at the beginning of the gripping process, and when the force reached a specific value, its increase tended to slow down. Each object had a different specific value, which was related to its size and mass; the larger the size and mass, the larger the specific value was. Moreover, the contact force pulling wire displacement curves for the four grasped objects as shown in Figure 10f are obviously different with the largest force measured during the gripping of a tomato. With the force–displacement curves obtained, we estimated which object was grasped in a prescribed situation based on the force feedback information. It was also observed that the angular displacement at the end of the gripper varied by the size of the object. Of the four samples taken in the experiment, the best shape adaptation and wrapping of the gripper was observed for the tomato.

### 3.3. Pinch Grasping

**Figure 11:**
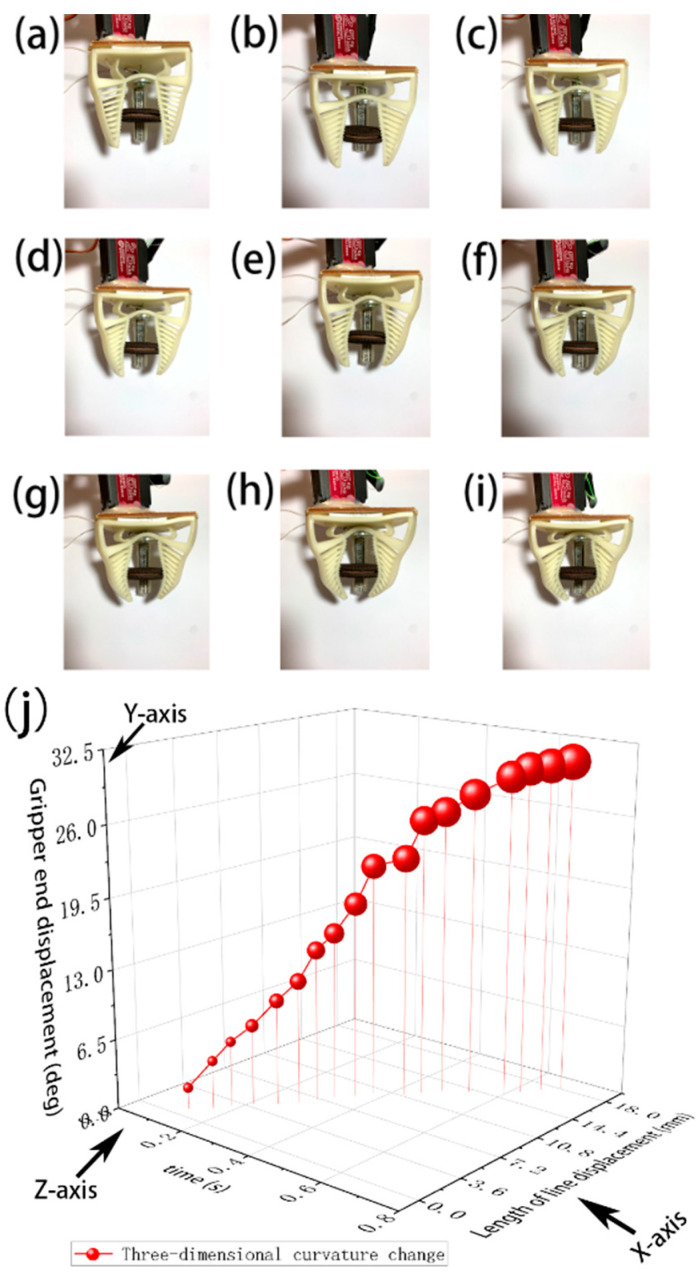
Examples (**a**–**i**) of a gripper grasping a biscuit. (**j**) shows a 3D scatter plot with color mapping, with the Y-axis being the angular magnitude of the gripper finger deformation, the X-axis being the length of the wire pulling displacement, the Z-axis being the change in time and the size of the bubble representing the magnitude of the contact force.

For some shapes, such as long, thin strips, wrapped-around gripping is not possible. Using pinch gripping instead, the following experiment demonstrated the effectiveness of this gripper when gripping such objects. For the food production industry, where the production and packaging of food cannot be automated and where most food products are prone to deformation or damage [27], the gripper would have potential application. In this test, a biscuit was pinched with the contact areas located in the middle section of the fingers, as shown in Figure 11a–i. The relationship among time, contact force, pulling wire displacement, and the finger end angular deformation are given in Figure 11j.

From the experimental result, the biscuit was not damaged and benefited from the shape adaptation of the fingers’ Fin Ray structures, demonstrating its feasibility and safety. The experiment also clearly showed that once the biscuit was sufficiently gripped, the increase in contact force became smooth after a specific value, even if the servo motor continued to pull the wire. Once this value was determined, it can be set to a given value for force feedback control, as shown in Figure 6, so that the entire soft robotic gripper system can work within this range for safe, reliable, and efficient food production application. The gripper design is well suited for use in the food industry and has a gripping force of around 0.1–3 N for small packaged foods and fruits.

### 3.4. Comparative Experiment

**Figure 12:**
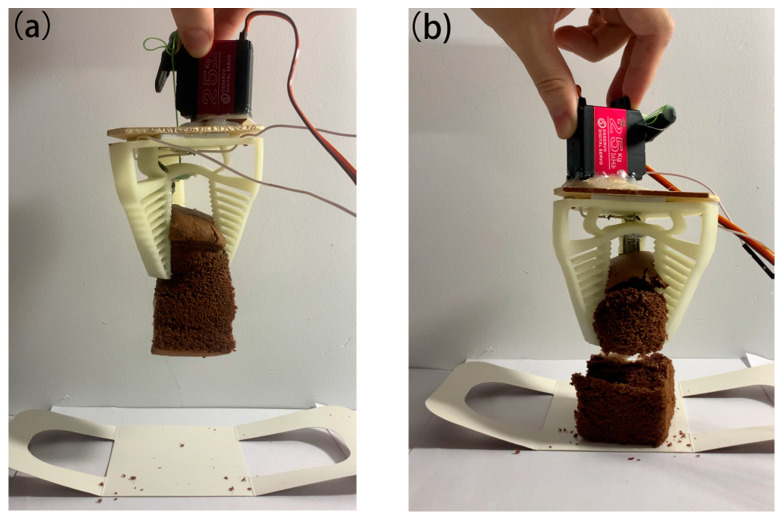
Comparative experiment diagrams of the proposed soft gripper grasping a soft and fragile cake with and without the force feedback function. Force feedback in action (**a**), force feedback not in action (**b**).

To demonstrate that the force feedback function improved the performance of grasping, a comparative experiment was conducted for the proposed gripper. In this experiment, a soft and fragile cake with a mass of 30 g was grasped twice. In the first case, the force feedback function was acting and the gripping force was set to 0.3 N. The gripping process followed the logic of the flow chart in Figure 6, and the servo will stop pulling the wire when the contact force achieves the given value such that the soft cake was not broken and, thus, a safe grasp was performed as shown in Figure 12a. In the second case, all the other test conditions were the same except the force feedback system was switched off during the grasping. The servo was set free to pull the wire and the soft cake was easily broken since the contact force information was unknown, as shown in Figure 12b. From this test, we determined that adding force feedback improved the gripper’s grasping performance, especially when grasping soft and fragile objects. Demonstrations of the gripper’s grasping performance are given in Supplementary Video S1.

## 4. Conclusion and Future Work

In this paper, a 3D-printed Fin Ray effect inspired soft robotic gripper with force feedback function was presented. Using 3D-printing technology, the overall gripper structure is lighter, easier to manufacture, less costly, and simpler to control than existing soft grippers inspired by the Fin Ray effect. With the pressure sensor embedded, the gripper’s grasping status can be monitored in real time and the gripper can be controlled to work according to predetermined expectations based on the force feedback.

**Figure 13:**
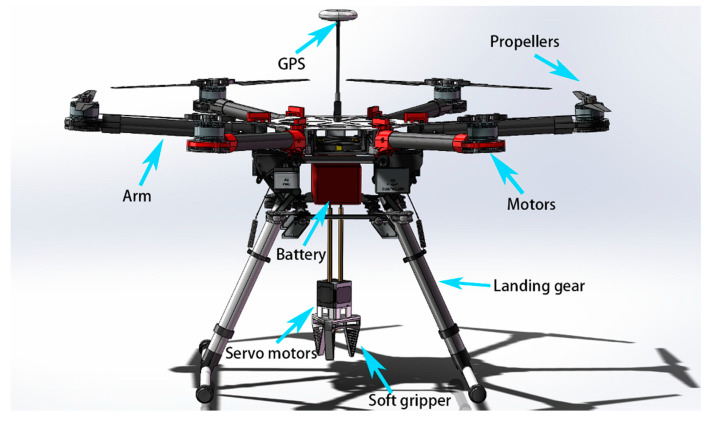
An intelligent delivery drone based on the proposed soft robotic gripper.

When in contact with an object, the fish fin inspired finger structure can passively adapt itself to fit the profile of the object. Thus, its inherent safety makes it suitable for human interaction in a wide range of scientific research and service applications. The soft material and elastic structure also compensate for excessive external forces and allow objects to be grasped without damaging them, making it ideal for non-destructive gripping. For grasping objects with various shapes in a production line, a single gripper can be applied to adapt to different shapes without the need to change or reprogram the gripper, thus saving time and cost. The gripper could also be integrated into intelligent assembly, automatic sorting, logistics, warehousing, and food processing lines. Figure 13 presents a possible application of the proposed gripper on a drone for delivery. Further improvements to the gripper can be made in future work, for example, optimizing the size of the fingers, adding more sensors to realize not only force feedback but also position feedback, etc., to suit a wider range of applications.

## Figures and Tables

**Table 1:** Comparison between the proposed flexible gripper and a commercially available gripper based on the Fin Ray effect.

	Gripper Weight (without Motor)	Fabrication	Force Feedback
The flexible gripper proposed in this paper	68 g	Fully 3D printed without assembly	With force feedback
Commercially available gripper [22]	200 g	3D-printed parts assembled with mechanical connections	Without force feedback

## Supplementary Materials

The following are available online at https://www.mdpi.com/article/10.3390/mi12101141/s1. Video S1: Grasping demonstration of the proposed soft robotic gripper with force feedback function.
